# A Patient-Centered Information System (myED) for Emergency Care Journeys: Design, Development, and Initial Adoption

**DOI:** 10.2196/16410

**Published:** 2020-02-25

**Authors:** Monika Westphal, Galit Bracha Yom-Tov, Avi Parush, Nitzan Carmeli, Alina Shaulov, Chen Shapira, Anat Rafaeli

**Affiliations:** 1 Technion - Israel Institute of Technology Haifa Israel

**Keywords:** technology, medical records, access to information, patient participation, electronic patient-provider communication, user-centered design

## Abstract

**Background:**

Medical care is highly complex in that it addresses patient-centered health goals that require the coordination of multiple care providers. Emergency department (ED) patients currently lack a sense of predictability about ED procedures. This increases frustration and aggression. Herein, we describe a system for providing real-time information to ED patients regarding the procedures in their ED medical journey.

**Objective:**

This study aimed to develop a system that provides patients with dynamically updated information about the specific procedures and expected waiting times in their personal ED journey, and to report initial evaluations of this system.

**Methods:**

To develop the *myED* system, we extracted information from hospital databases and translated it using process mining and user interface design into a language that is accessible and comprehensible to patients. We evaluated the system using a mixed methods approach that combined observations, interviews, and online records.

**Results:**

Interviews with patients, accompanying family members, and health care providers (HCPs) confirmed patients’ needs for information about their personal ED journey. The system developed enables patients to access this information on their personal mobile phones through a responsive website. In the third month after deployment, 492 of 1614 (30.48%) patients used *myED*. Patients’ understanding of their ED journey improved significantly (*F*_8,299_=2.519; *P*=.01), and patients showed positive reactions to the system. We identified future challenges, including achieving quick engagement without delaying medical care. Salient reasons for poor system adoption were patients’ medical state and technological illiteracy. HCPs confirmed the potential of *myED* and identified means that could improve patient experience and staff cooperation.

**Conclusions:**

Our iterative work with ED patients, HCPs, and a multidisciplinary team of developers yielded a system that provides personal information to patients about their ED journey in a secure, effective, and user-friendly way. *MyED* communicates this information through mobile technology. This improves health care by addressing patients’ psychological needs for information and understanding, which are often overlooked. We continue to test and refine the system and expect to find positive effects of *myED* on patients’ ED experience and hospital operations.

## Introduction

### Background

Emergency department (ED) care involves multiple assessments, tests, and treatments and engages multiple service providers, stakeholders, and resources [1]. The unpredictability and diversity of the medical state of ED patients poses operational and managerial challenges for sharing information with patients about their hospital ED journey. The lack of such information leads to helplessness and aggression in patients [2,3].

Technology is drastically changing health care delivery [4,5]. It facilitates physician support and patient monitoring, notably through electronic medical records [6,7] and dashboards [8]. Internet-based websites and patient forums increase communication between community clinics, patients, and health care providers (HCPs) [9-13], thus offering patients a wider scope of health and treatment information. Mobile apps are used to support patient self-monitoring, particularly for primary care (eg, medication reminders [14-17]). Novel technologies have begun to provide real-time, patient-centered information [18] for a series of medical care procedures, referred to as the *Patient Journey* [19-21]. The term is derived from the widely accepted concept of *Customer Journey* in Marketing literature, in which it refers to the activities and events included in service delivery from the customer’s perspective [22,23]. In this spirit, we are promoting a platform for informing patients about their hospital ED journey, to improve their understanding of the multiple procedures their medical situation requires.

Recently, Vorakulpipat et al [24] developed a system that shows patient status in real time, including waiting times, treatment locations, and treating teams. The system was developed for outpatient clinics and, therefore, also includes billing information and the number of people ahead in line. To reduce patient uncertainty, it presents an updated snapshot of the situation at any given moment. Similarly, Google developed a patent for the automated patient management system [25]; this enables patients to track their own status by viewing patient information on hospital servers at a kiosk or on their own mobile devices. In contrast to these two systems, *myED* reveals completed, current, and anticipated ED procedures, in addition to updated patient status. This is predicted to increase patients’ understanding of their personal ED journey.

### Objectives

Currently, patients in the ED depend completely on HCPs for information about their medical situation. However, HCPs do not have a systematic protocol for sharing such information with patients and are often working under time constraints. Hence, the communication of information to patients is frequently stalled and, typically, very brief. We searched for a means of communicating information about ED processes to patients and of reducing patient confusion *without* adding to the HCPs’ workload or delaying medical procedures.

*MyED* provides patient-centered information through a responsive website to facilitate access and support of all mobile devices*.* Accordingly, *myED* breaks down the complexity of ED care for each patient, building on the processing of existing electronic medical records. The system is designed to improve patients’ understanding of their personal ED journeys by means of a useful and user-friendly design, which ensures security and privacy [26,27]. Patients access a highly secure platform through a text message they receive to their personal mobile phone on ED admission. The system delivers information regarding individual patients’ ED *procedures* (eg, assessments, tests, and treatments) and associated waiting times.

**Figure 1:**
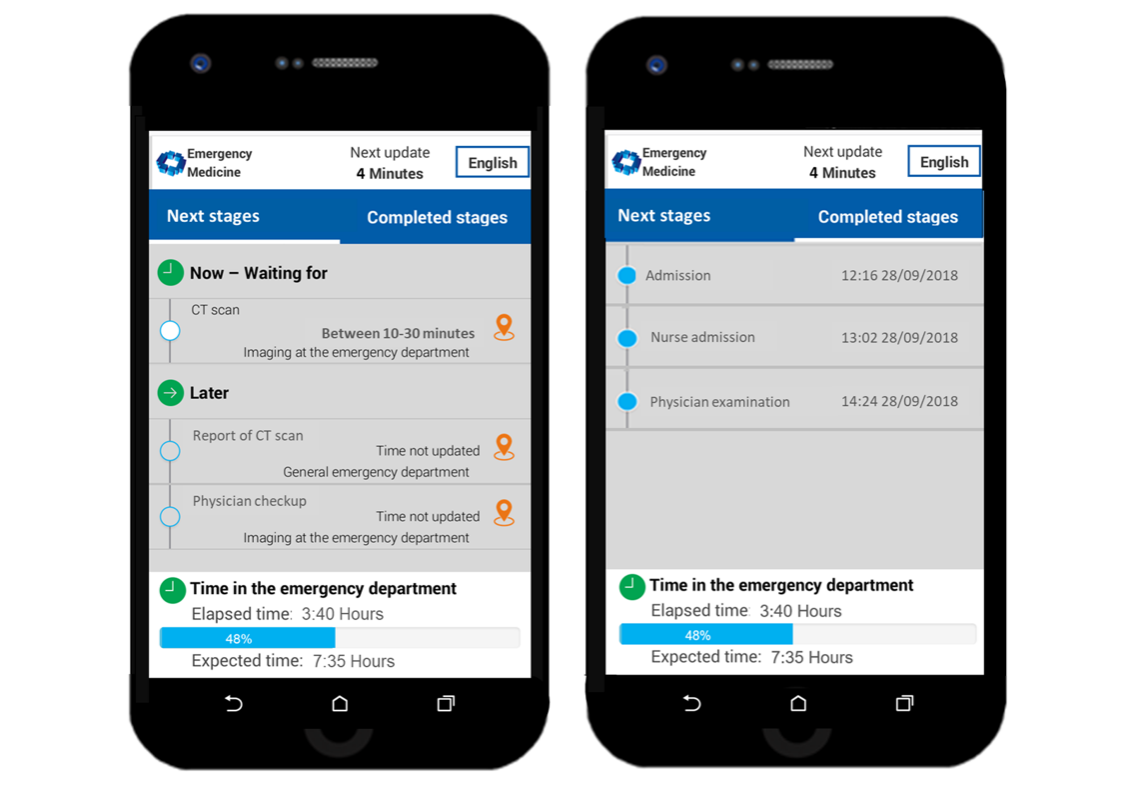
Screenshots of *myED*, available to an actual patient on September 28, 2018, 15:56.

Our design and evaluation of *myED* integrates key elements of the technology acceptance model (TAM; Davis et al [28]) and the information system (IS) success model [29,30]. We used the two key variables *perceived usefulness* and *perceived ease of system use* as guides in our evaluation process because they are known to be valid predictors of attitudes toward system use [28], actual use, and user satisfaction [29,30]. Figure 1 shows screenshots of *myED,* which present an actual patient’s journey.

The primary objective of this line of research is to validate effective information communication to patients through their mobile phones and to increase patient satisfaction through an enhanced understanding of their personal ED journey. The goal of this paper was to report on the design, development, and initial evaluation of *myED*.

## Methods

### Overview

**Figure 2:**
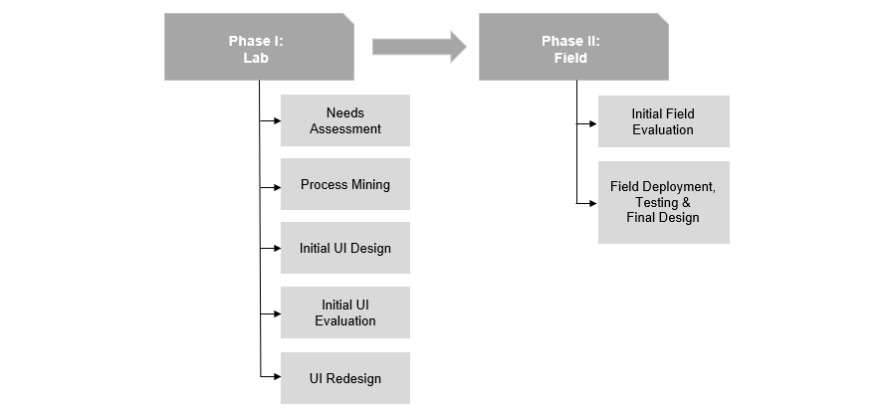
Overview of the methods used to design, develop, and evaluate *myED*. UI: user interface.

The research was conducted in collaboration with an ED of a medium-sized (477-bed) tertiary hospital. The study was reviewed and approved by the Institutional Review Board and conducted in accordance with the Declaration of Helsinki. Phase I included assessing patient needs, using process mining [31,32] to dynamically create patient information, designing an initial user interface (UI), conducting laboratory evaluations of this design, and redesigning the UI. Phase II comprised deploying and testing the system, identifying barriers to adoption, and refining the design accordingly. The methods and results are reported separately for the two phases, as illustrated in Figure 2.

### Phase I

#### Needs Assessment

We conducted semistructured interviews with 2 ED patients, 4 family members, and 5 HCPs to assess patients’ needs for information about their ED care*.* This sample size follows the data saturation criteria for qualitative research (see Sandelowski [33]). The following questions roughly guided interviews with patients and family members: What information did you receive about what is going on with your ED care? Have you tried to find out what is going on? Who did you talk to in the ED? Do you understand why you are waiting and whom you are waiting for? Do you know how much time you will have to wait? What information may be helpful to you at this time? HCPs were asked about *their* perspectives on these patient-related questions.

#### Process Mining: Mapping Patient Journeys and Predicting Waiting Times

We used *process mining* [31,32] tools (ie, *process discovery* and *queue mining*) to mine patient-related information stored in the medical databases of the hospital ED. We accessed all available information of patients in the ED for 39 months (2014-2017).

**Figure 3:**
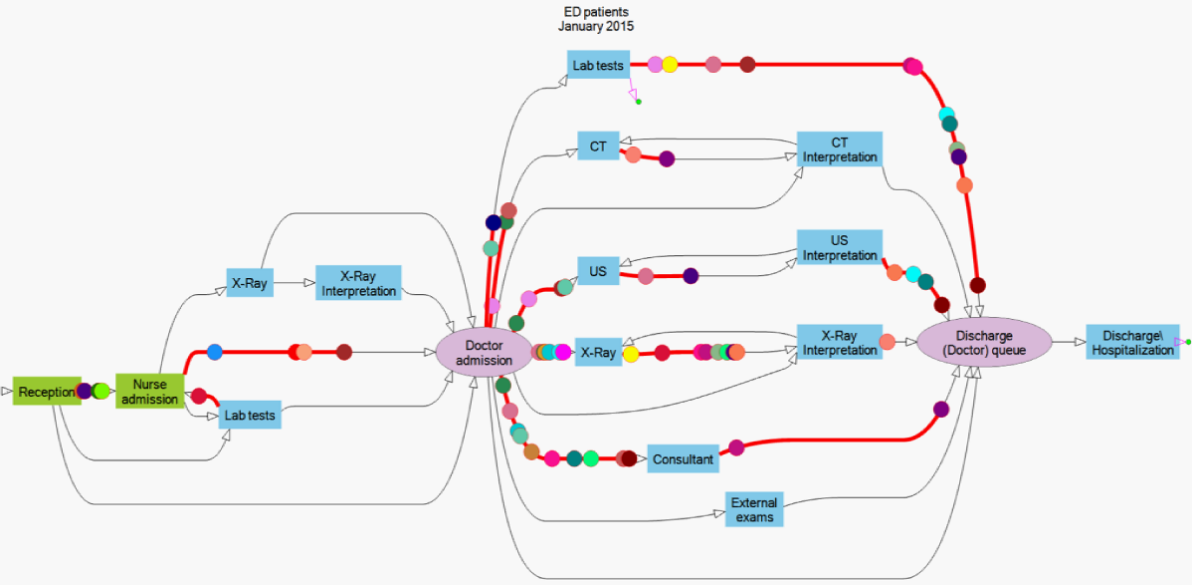
Process chart of all possible emergency department patient journeys.

First, we mapped all possible patient ED journeys. Using process discovery tools [34], developed in the *Technion Service Enterprise Engineering (SEE)* Lab [35], we decoded the medical procedures in each archived patient’s medical record. We then aggregated these data across all patients and visualized this aggregation as a process chart that shows all possible ED journeys (see Figure 3). This information enabled building real-time techniques that detect individual patient journeys and dynamic updating of information during the ED stay.

Second, we developed a means of estimating individual patient waiting times for each specific ED procedure. Following Ang et al [36] and Carmeli et al [37], we incorporated queuing theory–based results as features in machine learning methods. We started by estimating the workload in each procedure of ED care. For example, we calculated the number of people queued for a computed tomography (CT) scan when a specific patient entered this queue, and the service rate of the CT scan (ie, the number of patients who undergo a CT scan per hour). We trained a machine learning model (eg, random forest [38]) to predict waiting times for each patient regarding each procedure. The learning model comprised the following types of variables: (1) *time* variables: hour of day, weekday; (2) *patient static* variables: triage level, arrival type (eg, ambulance, walk-in), age, gender; (3) *patient dynamic* variables: completed and anticipated procedures in the patient ED journey; and (4) *dynamic workload* variables: queue length, service rate, the time waited by the last patient to receive treatment, and the total number of patients in the ED.

#### Initial User Interface Design

Parallel to mining hospital information, we translated some incomprehensible language of medical information into lay terms. This was consequent to the review of a large sample of medical records that identified confusing or unclear medical terms (eg, hemoglobin level and white blood cell count). In consultation with hospital staff, we identified appropriate substitute lay terms. We further verified the clarity of the terms with patients during the first few days of system deployment and did not find any problems regarding the comprehension of the text.

We developed an initial UI for communicating the relevant patient-centered information; our aims were usefulness and ease of use. We included three UI views of ED journeys: *Completed steps* (procedures a patient already completed); *Now* (procedures for which a patient is currently waiting); and *Future steps* (anticipated procedures). The *Now* view shows an estimate of the waiting time for the current procedure, and all views show an estimate of the patient’s total length of stay (LoS). Time estimates are updated every 5 min, based on changes in ED load and patient prognosis.

#### Initial User Interface Evaluation and Redesign

**Figure 4:**
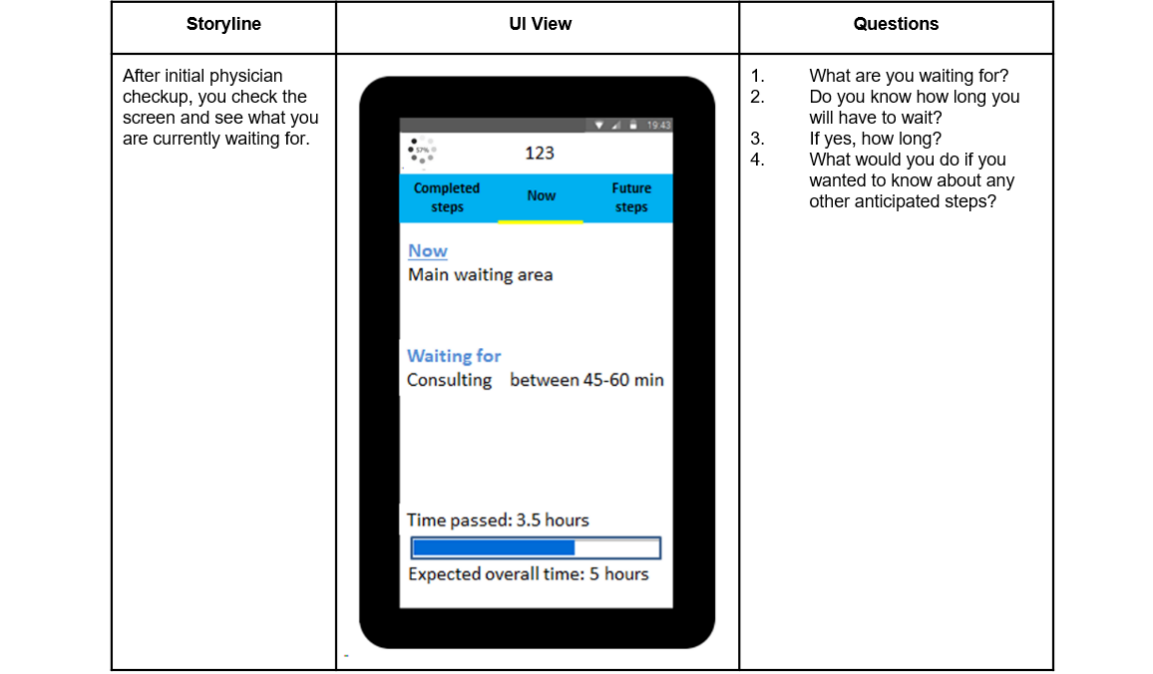
Sample storyline, screen, and questions used to test the initial user interface design.

We ran a study to evaluate the understanding of the information communicated by our three UI views. We recruited 255 participants on PanelView [39], an online platform for creating surveys and recruiting participants to take the surveys. We asked them to imagine arriving at an ED and being informed about their ED journey through a novel system that they access through their own mobile phone. The three static UI views were embedded in a three-part storyline. Participants were led through these three screens as depictions of the scripted ED journey and then responded to questions about what they saw. Figure 4 illustrates the *Now* part of the storyline; each participant saw similar views for *Completed steps* and *Future Steps*. On the basis of the initial UI evaluation, we revised the UI design, as described in the Phase I Results section.

### Phase II

#### Initial Field Evaluation

We deployed *myED* in the pediatric section of the hospital’s general ED. This enabled a pilot in a smaller, more controlled environment. The 6-month pilot identified issues and potential obstacles arising from the mining process.

#### Field Deployment, Testing, and the Final Design

After initial UI evaluation and redesign, we deployed *myED* in the ambulant adult section of the same ED. The ED includes all disciplines except maternity and otolaryngology. The adult section is divided into three subsections: ambulant (triage score 3-5), lying-in (triage score 2-3), and trauma (triage score 1).

As part of the routine ED admission process, patients were asked to provide their phone numbers. They were then informed about *myED* and sent a text message with a link to the log-in screen. After agreeing to the terms, including their consent to be part of a study on improving patient ED experience, they could enter the website anytime with their details. We designed *myED* to impart a high level of security and protection of privacy, thus mirroring the hospital’s ED medical records and to extract only information about the patients’ medical procedures. The system’s architecture is based on a demilitarized zone (DMZ) server that is separate from the hospital databases; *myED* has access to the DMZ server only, adding an additional layer of security. Nonsensitive patient information is extracted and displayed on the patient’s mobile phone; no confidential information is displayed on the screen, and patients are not identified in the *myED* records.

As part of the system evaluation, we assessed the reactions of patients and HCPs and made final small changes to the system design.

First, 5 students who served as research assistants (RAs) shadowed and interviewed a sample of 482 patients for 2 hours a day during the first four weeks after deployment (July-August 2018) to understand the perceived usefulness and attitude toward *myED* use. RAs approached ED patients one-by-one (excluding those who seemed to be in great pain) and asked if they had entered *myED* and if they were willing to share their feedback. If a patient had not yet entered *myED*, the RA asked if he or she was interested in doing so. If relevant, the RAs helped with the entry process; if not, the RA asked if the patient was willing to share why he or she did not want to use *myED*. Specifically, we measured four types of attitudes toward *myED* use: (1) self-initiated entry to *myED*, (2) *myED* entry initiation once approached, (3) inability to enter *myED*, and (4) disinterest in entering *myED*. We also noted perceived usefulness from *myED* users (eg, “I really like this system. Finally, someone cares about the patients!”) and nonusers (“I have my file in my hand, I don't need your system!”).

Second, we analyzed *myED* records for actual system use during 3 months of system deployment (August-October 2018). We excluded patients who did not receive a text message (888/4767, 18.63%: eg, their phones were not with them or were without battery power or internet connection). This decreased the baseline relevant population size to 3879. We then computed the number of people who used *myED* during August-October 2018 (1131/3879, 29.16%) and identified the point of their ED journey when they first logged in. We also reported the system adoption rates that we reached in the third month of deployment (October 2018): 1614 people received a text message (81.39% of 1983 who arrived), of whom 492 (30.48%) used *myED*.

Third, 5 RAs (students) surveyed a sample of 349 people about their understanding of the personal ED journey, both before and after system deployment. RAs approached ED patients one-by-one (excluding those who seemed to be in great pain) and invited them to participate in a survey regarding the ED service. In June 2018, 60.2% (210/349) of people responded (system nonusers, the *control group*), and in August-October 2018, 39.8% (139/349) responded (system users, the *intervention group*). Short surveys assessed patient understanding (“I understand the sequence of procedures of my treatment”; “I understand the various stages of my treatment,” on a scale from 1 [not at all] to 7 [very much]), the time they had already spent in the ED until they filled out the survey, and patient demographic characteristics (age; gender; economic status, defined as the number of people divided by the number of rooms at home; religion: Jewish, Muslim, Christian, Druze, other).

Fourth, we interviewed 5 HCPs regarding their own attitudes and their perceptions of patients’ attitudes of the usefulness and ease of use of *myED*. We followed a semistructured interview protocol (eg, Have you seen *myED*? Do your patients use it? How do you feel about *myED*? How do you think patients feel about *myED?* Does *myED* influence your work? Does anything bother you about *myED*? Any ideas on what could improve *myED*?).

## Results

### Phase I

#### Needs Assessment

Interviews strongly underlined patients’ need for information about their medical procedures (what, when, where), and about waiting times (when, how long). All 11 respondents mentioned all these issues. Hence, *myED* was developed to address the following needs: information about (1) procedures in the ED journey, (2) estimated waiting times, (3) the location for each procedure (because some procedures occur outside the ED, eg, in outpatient clinics), and (4) total ED LoS.

#### Process Mining: Mapping Patient Journeys and Predicting Waiting Times

*MyED* generates individual, constantly updated information, fed to the mobile phone of patients (see Figure 1). The system translates information stored in hospital ED medical records to patient-friendly information and updates itself every 5 min, using the following analyses:

#### Process Discovery: Identifying Procedures in a Patient Journey

We aggregated detailed medical examinations that are conducted at the same time and location into operational procedures. For example, hemoglobin level and white blood cell count (which are recorded separately in the ED medical records) were aggregated into *lab tests* because patients experience them together and view them as a single procedure. Aggregating information across all patients produced all possible ED journeys, as depicted in Figure 3 (for a dynamic view, see [40])**.**

**Figure 5:**
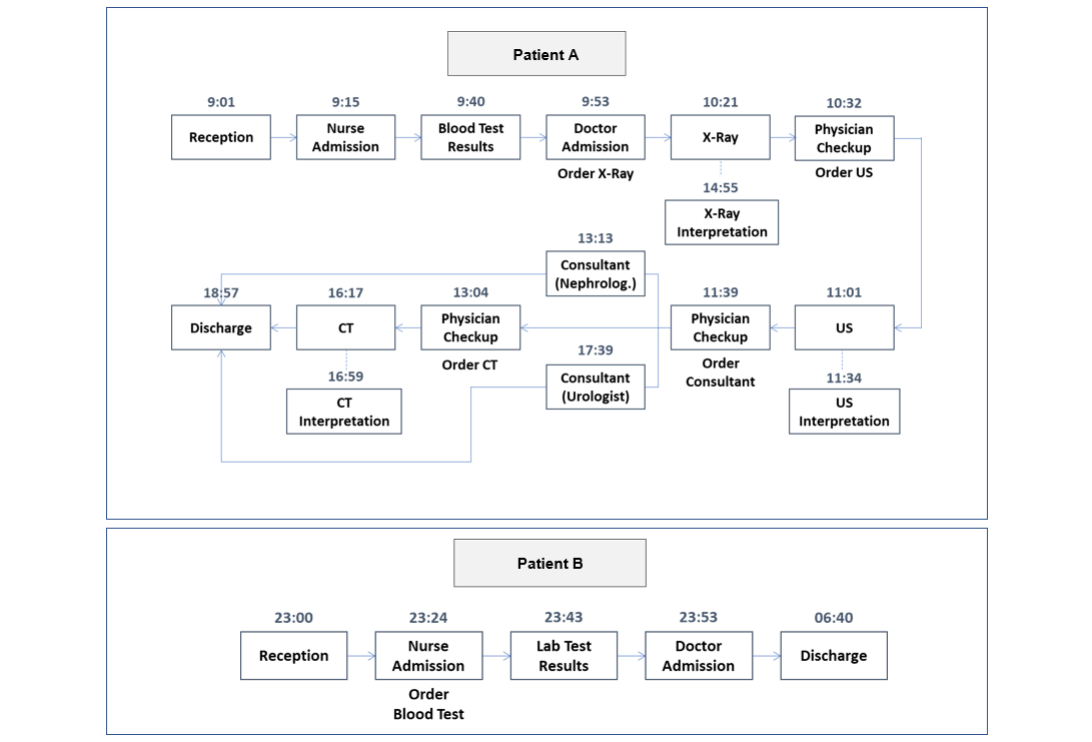
Two patients’ emergency department journeys identified using process mining. CT: computed tomography, US: ultrasound.

Each patient’s journey is depicted as a distinct path in this graph, comprising a certain order of medical procedures. There is almost no predetermined order, and patients can simultaneously wait for two or more ED procedures, with no clear indication of which should be performed first. Patient journeys also vary in complexity. For example, Figure 5 shows a more and a less complex journey (Patient A and Patient B, respectively). The full complexity of a patient’s journey evolves continuously during the patient’s ED stay. Therefore, *myED* constantly adjusts information communicated to patients, based on updates to ED medical records.

We also identified procedures that typically occur sequentially; for example, ultrasound (US) examinations are always followed by US interpretation. We use such identified sequences to predict elements in the ED journey before they appear in the ED medical records. This is the foundation for building the information *myED* presents to patients as *anticipated procedures*.

#### Queue Mining: Estimating Waiting Times

ED medical records include completion times for ED procedures and are the source of the waiting times presented in the *Completed steps* view of *myED*. Queue mining methods enable *myED* to present patients with waiting times for anticipated procedures. Following Carmeli et al [37], we identified a probabilistic range of waiting times for each procedure. We used the 0.15-quantile as the lower and the 0.85-quantile as the upper bound of the reported range, which ensured that waiting times of no more than 15% of the patients exceeded our prediction.

#### Initial User Interface Evaluation and Redesign

In total, 255 participants (age: mean 53 years, SD 8.6 years; 140/255, 54.9% female) evaluated the three UI-view design in the online study. The results showed that a high proportion of participants (202/255, 79.2%) indicated understanding the presented information. However, responses to specific questions about the anticipated procedures showed that 29.8% (76/255) were confused by the separation between *Now* and *Future Steps* views. Hence, we redesigned the UI into two views: *Next stages* and *Completed stages*, as depicted in Figure 1. *Next stages* includes both upcoming procedures (*Now – Waiting for*) and subsequent procedures (*Later*). Hence, the *Now* and *Future steps* were collapsed into one view.

We found that people prefer that waiting times are presented as a range (*between X and Y min*). Most participants (151/255, 59.2%) reported that this presentation seems more reliable and trustworthy than *at least X min* (60/255, 23.5%) and *about X min* (44/255, 17.3%). Figure 1 illustrates this. The patient depicted needs to wait 10-30 min for a CT scan. The same view shows that later he or she will wait for a report of the CT scan and meet a physician (Figure 1, left side). The *Completed stages* view (Figure 1, right side) shows that the patient was admitted (at 12:16 PM) and already saw a nurse (at 13:02 PM) and a physician (at 14:24 PM).

### Phase II

#### Initial Field Evaluation

The 6-month pilot in the pediatric ED identified several issues and potential obstacles arising from the mining process. First, hospital databases are not always updated by the medical staff in real time. This creates gaps between the actual patient journey and the information available in *myED*. For example, we found that for 74.74% (1098/1469) of the patients, laboratory tests were reported in the databases only *after* completion. Hence, many patients could not see any information in *myED* regarding these laboratory tests while they were still waiting for them. Similarly, some procedures cannot be accessed by *myED* because they are recorded in inaccessible databases. For example, *myED* cannot access outpatient clinic databases. According to the data we gathered during April 2014-March 2015, this occurs for 0.26% (50/19,279) of the patients. Missing updates of delays also create a statistical challenge as they create missing data in the model that predicts waiting times and LoS. The field evaluation revealed inaccuracies in predictions, which were handled by retraining our learning models. For example, our algorithm underestimated 47% (46/98) of the waiting times for US examinations before the retraining (July 2018), and only 22% (17/76) afterward (August 2018).

Second, hospital guidelines change over time, creating changes in patient journeys, and, if not updated, inaccuracies in *myED*. For example, in the historical data used to create the system, all x-ray tests were analyzed by a radiology specialist. However, during *myED* deployment*,* new guidelines allowed regular physicians to analyze some simple x-ray tests. This created mismatched information because *myED* informed patients they were waiting for an x-ray interpretation, instead of a physician. We quickly adapted *myED* to such guideline changes. This example emphasizes the need to continually update the system to reflect policy and protocol changes and to evaluate system accuracy periodically.

Third, the unpredictability of some ED procedures means that the actual waiting time for a specific ED procedure may exceed the waiting time predicted by *myED*. For example, a meeting with a specialist may be delayed substantially because of an emergency in his or her home unit. This creates a dilemma in deciding what information to show patients when the actual waiting time exceeds the upper bond (85th-quantile) reported. We considered three options: (1) show a generic estimate such as *up to 15 min* until a procedure is completed; (2) stop showing information, noting *time estimates are not available*; or (3) tell patients to check with HCPs regarding the delay. Consultation with hospital staff identified the first option as the best.

#### Field Deployment, Testing, and the Final Design

During the first month of deployment, 482 patients were actively approached (age: mean 50 years, SD 18.9 years; range 20-90 years; 265/482, 55.0% female) to use the system. Of them, 19.9% (96/482) were already using it, and 40.1% (193/482) then agreed to use it.

Of 349 respondents, 49 (11.7%) provided incomplete demographic information and were, therefore, excluded from the analysis that assessed patients’ understanding of their personal ED journey. The intervention group (*myED users*) comprised 139 respondents, and the control group 210 respondents. Patient age was similar between the groups (age: mean 46 years, SD 16.1 years, range 18-83 years; age: mean 46 years, SD 17.9 years, range 18-93 years, respectively). Sex distribution was also similar (51.9% and 51.8%, respectively). The patients in the intervention group had a significantly better understanding of their ED journeys than did the patients in the control group after controlling for their age, gender, economic status, and religion (*F*_8,299_=2.519, *P*=.01).

Of the patients actively approached, 39.2% (189/482) provided open-ended responses about their attitudes toward system use. All following responses of patients and medical staff were coded as [X.Y, gender, age], with 'X' representing the interviewee number and 'Y' the date in 2018 in ddmm format. Positive responses (118/189, 62.4%) included short praises (eg, “good!” “nice!” and “great!”), and general delight:

I used it all day long! It's good.

311.0908, female, 28 years

This is something new, good idea.

112.2607, male, 47 years

A very effective system, thank you.

326.1908, male, 40 years

The system is excellent.

297.1608, female, 35 years

All 5 HCPs interviewed also perceived *myED* as both useful and easy to use and agreed with its potential benefits for both patients and medical staff:

The staff should accept [the system] as an inseparable part of handling patients here. There were some patients who came to us and didn’t know how to activate the system, but the procedure is very simple; it is quite friendly and easy to use.

It is very effective, it's a shame that it wasn’t there before. It really helps us, people really check and come to me and say they see their tests.

1.0708, male, 40 years

I hope it will help both patients and us, as it will enable them to understand their treatments.'

It will definitely do no harm, it can only be useful.

5.0708, female, 48 years

#### Barriers to System Adoption and Corresponding Modifications

In the first week of deployment in the ambulant adult ED, *myED* log-in rates varied greatly (ie, between 7/54, 13% on day 3 and 27/64, 42% on day 1). We noticed several causes of poor system adoption.

Initially, admission staff were required to offer *myED* to patients and to ask for consent before manually registering them. One of 5 HCPs expressed concerns about delaying medical procedures:

I worry that the system will add time until patient triage, and that we will lose critical time-to-triage, which must be done within 15 minutes.

5.0708, female, 48 years

We saw that HCPs were not offering *myED*, especially when the ED was loaded, to not delay time-to-triage. Another cause of poor adoption was the identity format required to log into *myED*, which was designed according to the format used in ED medical records. The design was different from the routinely used identity number. This confused patients and stalled the use of *myED*. We handled both issues with a *login page redesign*. Specifically, we introduced automatic registration, thus integrating patient consent into the log-in process and modified the format of patient identification. This yielded log-in rates of 26.26% (266/1013) within 1 month of system deployment.

Second, not all patients knew about *myED.* We introduced *local advertising of myED* within the ED; flyers about the system increased log-in rates by 13.44% to 29.79% (373/1252) in the second month of system deployment.

Third, people arriving at the ED were often preoccupied and stressed and frequently missed the text message that was automatically sent for logging into the system:

I have such a migraine I can't look at anything.

128.2607, female, 55 years

I don’t have the patience for this now.

129.0808, female, 38 years

I can’t listen to what you say, I don’t feel well.

90.0608, male, 35 years

I’ll look at it at home after all this is over. I don’t feel well right now.

31.2407, male, 70 years

Fourth, *myED* reports of 266 users (August 2019) showed the mean time-to-first-entry as high as 60 (SD 74) min after arrival. We, therefore, introduced a new *reminder text message*, sent 30 min after arrival to anyone who had not yet logged in; 30 min allow most patients to complete the initial triage, nurse admission, and first physician examination. This shortened time-to-first-entry in the following 2 months by 18%, to 49 (SD 62) min (865 users, September-October 2019). 

**Figure 6:**
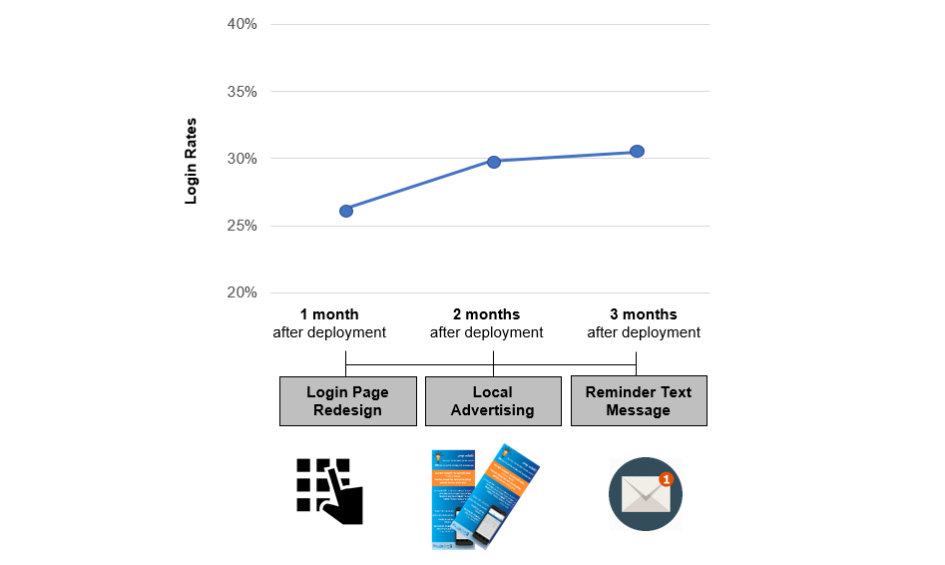
Influence of design modifications on myED login rates.

Figure 6 summarizes the effects of these design modifications, which further increased log-in rates by 2.32% to 30.48% (492/1614) in the third month—an impressive adoption rate in such a short time [41].

**Figure 7:**
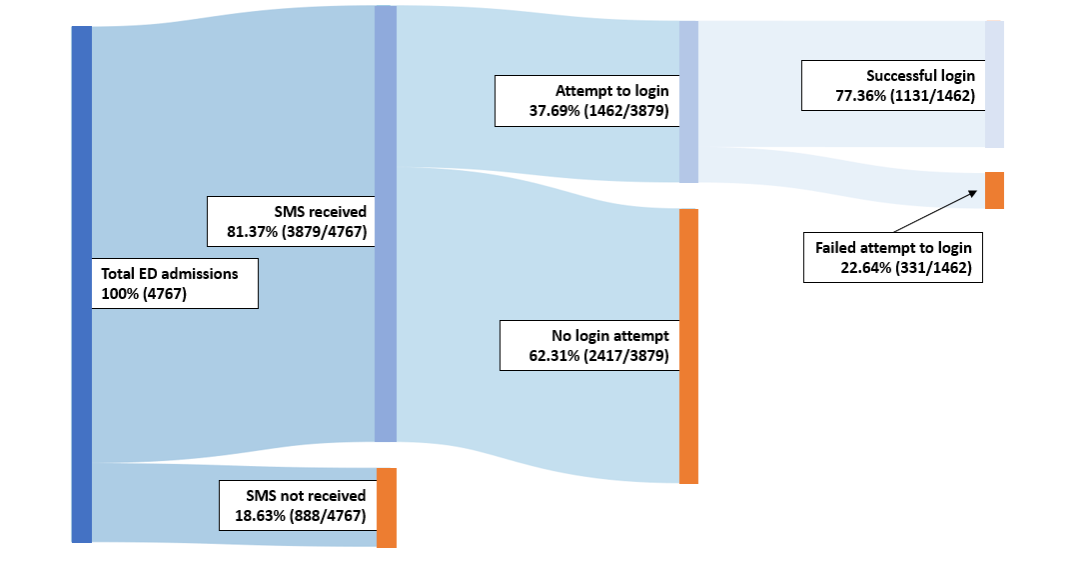
System adoption in first 3 months of deployment (08-10/2018). ED: emergency department.

Further barriers to *myED* log-in attempts included issues with people’s phones, which precluded their receiving the text message (888/4767, 18.63%). As depicted in Figure 7, 81.37% (3879/4767) of patients in the ED received the text message, of whom 37.69% (1462/3879) attempted to log in. Log-in failures (331/1462, 22.64%) included technical issues such as disabled cookies.

Of 189 patients who provided open-ended responses about their attitudes toward system use, 37.6% (71/189) relayed negative or mixed (both negative and positive) attitudes. In addition, 14% (10/71) alluded to technology literacy or dependence on others as an issue:

I am still in the Stone Age with regard to anything digital.

94.2407, female, 80 years

I talk to, and text only with my children.

71.0107, male, 70 years

I trust my wife will take care of everything.

12.1807, male, 40 years

Most challenging to system acceptance were doubts about the value of *myED* (49/71, 69%):

I have my file in my hand, I don't need your system.

20.1807, female, 20 years

Why do I need this if the doctor will tell me the results of the blood test?!

223.0708, female, 65 years

These reactions seem to be related to the long and frustrating time spent in the ED:

What for? I know what to expect…I know what I am waiting for and for how long - basically, all night.

48.2607, male, 60 years

It serves no purpose if I have to wait for 4 hours.

96.2407, female, 79 years

This is worthless! I am waiting for the cardiologist and I don't care that this is what it says on my mobile's screen! What I care about is that I've been waiting here already for 5 hours!

316.1208, female, 60 years

However, longer LoS did not hinder system adoption (August-October 2018). On the contrary, *myED* users had longer mean LoS than nonusers (4.5 and 3.9 hours, respectively).

Ten percent (7/71) of the comments we received were related to the accuracy of *myED* information:

Worthless! I am half an hour past my neurological consultation and it says here I am waiting for it!

13.1807, male, 20 years

I'm not waiting for the procedure it says!

268.1208, male, 50 years

Two of 5 HCPs initially expressed concerns about *myED* and their workload:

I don’t feel it will reduce the workload. They [patients] still come to ask what is going on, and what they are waiting for.

Another thing that we feared could happen is that there will be more nagging once they receive information that results arrived.

5.0708, female, 48 years

Once test results arrive and the patient knows it, it will only put more pressure. They [patients] can start knocking on doors, and this will put pressure on [other] patients and us.

3.0708, male, 39 years

These concerns suggest that merely informing patients about procedures could potentially increase HCPs’ workload, if not done appropriately. However, when asked whether these concerns materialized, HCPs responded that they did not. Rather, patients do not seem to ask more, but simply ask different questions:

I feel that patients’ questions have changed. Now they already know that the test results have arrived.

2.0708, female, 51 years

#### Suggested Improvements for the System

Eleven percent (21/189) of the patients who responded to open-ended questions suggested ideas for improvement. For example, they asked why the system does not provide more detailed personal or medical information:

After logging in there should be some identification that this is indeed my account, like my name or identity number, on the screen.

211.0608, male, 80 years

I would have liked to see results of tests, like I can see in the HMO website.

219.0608, male, 50 years

However, owing to security concerns, identifying patients and providing medical information would require an even higher level of security in the system, which would substantially complicate registration. We were concerned that this would hamper people’s willingness to adopt a system that they can use only for a few hours. Showing test results can also be risky, as 2 of 5 HCPs noted, for example:

I was afraid that the system would send the actual results of the tests...I know from other HMOs [health maintenance organizations], that if people suddenly see that the result of a blood test appears in red, they are stressed. It can be very stressful [for us] in the ED.

3.0708, male, 39 years

Abnormal test results can add to patient stress, whereas normal test results may cause patients to leave without being seen*—*both are undesirable consequences.

Patients also asked for the names of the HCPs they were waiting for:

The system does not give me a lot of information, I suggest to add the doctor's name in the relevant step.

109.2607, male, 50 years

Providing this information in *myED* is impossible because it does not appear in ED medical records.

## Discussion

### Principal Findings and Comparisons With Previous Work

This paper describes the development and initial implementation of *myED*, a system that addresses the need of ED patients for information about their medical journey. *MyED* is a personalized, frequently updated information system, accessible by patients anywhere and anytime during the ED visit, on their personal mobile phones. Vorakulpipat et al [24], who developed a system comparable with *myED*, focused on outpatient clinics. Although their system provides additional information, such as the number of people ahead in line and the name of the treatment team, only a patient’s current status is revealed. In contrast, *myED* reveals the entire patient ED journey. The underlying assumption is that comprehensible, continuously updated information about personal ED journeys will improve patients’ understanding of the process and reduce frustration and anger [42]. Our results attest to increased patient understanding and overall positive responses to the system. The adoption rate of *myED* at the end of the first 3 months was satisfactory (492/1614, 30.48%), thus confirming the viability of the system.

Our design and evaluation of *myED* integrates key elements of the TAM [28] and IS success model [29,30]. Specifically, we report positive perceptions by patients and HCPs of ease of use and usefulness, positive and negative attitudes toward use, and a reasonable proportion of actual use of *myED*. The mixed methods approach enabled presenting multifaceted data of *myED* users and nonusers, as well as of HCPs regarding these cognitive and behavioral aspects of *myED* use.

### Meeting Challenges

Our study offers ways to tackle three distinctive challenges that we encountered: (1) extracting real-time ED medical records and transforming them into comprehensible and accurate information, (2) providing information to patients without disrupting the ED workflow [43,44], and (3) getting patients to use their personal mobile phones to obtain information during ED visits, which are short-term and have a limited user-engagement period (a few hours, during the day of their visit).

To address the first challenge, we modeled ED patient journeys and presented them to patients, using an innovative, unique combination of operations research tools (ie, process discovery and queue mining [31,32]) and user-centered design methods. The interdisciplinary effort enabled translating medical and process-related information in ED medical records into real-time information regarding personal procedures. Specifically, *myED* translates existing but fragmented information into clearly structured information regarding completed, current, and anticipated medical procedures, including estimated waiting times.

Meeting the second challenge, *my*ED works with minimal disruption to the ED workflow. First, the system relies on available information, extracted directly from ED medical records that HCPs routinely update. Second, patients do *not* see actual test results or other concrete medical information. Such information requires professional interpretation and can cause patient anxiety if not communicated appropriately. Third, the *myED* design enables automatic log-in and thus ensures that the registration process does not increase time-to-triage. Fourth, the *myED* design affords patients independence by requiring no HCP involvement. The intuitive *myED* design is easy to use and employs lay terms (not medical jargon), reducing patient confusion. Finally, *myED* provides location information, which has thus far been provided only by HCPs, if at all. This improves patient orientation and can thereby reduce delays.

Regarding the third challenge, *myED* was well accepted and adopted by patients despite the short duration of user-system engagement. Mobile technology increasingly provides patients with health care information; we showed it can also be useful in the ED. As *myED* users are mostly one-time visitors, the increase in system adoption reflects the success of *myED* design modifications.

### Limitations

A number of limitations are relevant to the conduct of this study. First, the number of persons interviewed for the needs assessment was small (11 in total). In addition, the cohort of users of *myED* may reflect a selection bias. One reason is that RAs who encouraged the use of the system were instructed not to approach patients in great pain. Moreover, we only deployed and tested *myED* in the pediatric and ambulant adult ED of one specific hospital. Patients in the lying-in unit, or in the same units in smaller or larger hospitals, may have needs that were not identified in this research.

### Future Work

The accuracy of *myED* depends on regular updates of medical information by HCPs. Otherwise, discrepancies may occur between the information presented in *myED* and the information patients obtain from other sources. Such discrepancies can reduce patient trust and—rather than offering relief—exacerbate patient confusion. Ways to avoid such discrepancies must be sought, without disrupting the ED workflow and HCP workload. For example, updating information during patient meetings on portable computers could improve real-time information, without adding ED workload. Second, the quick and short-term user-system engagement of *myED* is a continuous challenge. An effective system would avail rapid acceptance and adoption beyond what we accomplished. Low system adoption among patients who feel sick or are in pain, and among those who are technologically illiterate, remains a challenge. In the effort to improve system adoption, the *onboarding process*, (ie, receiving and opening the link in the text message, logging in, relogging) should be further simplified. Third, a new challenge we identified during *myED* implementation is the need to secure the stability of the fragile relationship between ED staff and patients. HCPs recognized the benefits of *myED* but expressed concerns that it could change the power relations between staff and patients if patients see information *before* the staff [45]. To avoid such concerns, we must strive for mutual HCP-patient empowerment (see Parush [46]).

Research has shown that providing information about waiting time affects people’s behavior [47]. Our design includes waiting times in range format (eg, *between 30 and 45 min*) to decrease the risk that patients would be unavailable once they reach the head of the line. Future research is needed to verify this prediction. Inaccuracy of waiting time estimates has been shown to decrease trust and increase frustration [48-50]. Hence, future research should continue to explore the optimal means for presenting waiting times to ED patients. Finally, another direction for future research is the investigation of patient-related measures beyond patient understanding. These include patient satisfaction of the ED visit and experienced frustration, as well as other important operational measures, such as a patient leaving without completing the planned procedures [51].

In summary, our research shows that *myED* is a novel and revolutionary approach for improving a patient’s understanding of his or her personal ED journey. This new use of ED medical records can improve patients’ experience of ED visits.

## Abbreviations

CT: computed tomography
DMZ: demilitarized zone
ED: emergency department
HCP: health care provider
IS: information system
LoS: length of stay
RA: research assistant
TAM: technology acceptance model
UI: user interface
US: ultrasound
